# Vaccination with a combination of STING agonist-loaded lipid nanoparticles and CpG-ODNs protects against lung metastasis via the induction of CD11b^high^CD27^low^ memory-like NK cells

**DOI:** 10.1186/s40164-024-00502-w

**Published:** 2024-03-29

**Authors:** Alaa M. Khalifa, Takashi Nakamura, Yusuke Sato, Hideyoshi Harashima

**Affiliations:** Faculty of Pharmaceutical Sciences, Hokkaido University, Kita-12, Nishi-6, Kita-ku, Sapporo, 060-0812 Hokkaido Japan

**Keywords:** STING agonist, CpG-ODN, CD11b^high^CD27^low^ memory-like NK cells, Prophylactic cancer vaccine, Drug delivery system

## Abstract

**Background:**

Natural killer (NK) cells are effective in attacking tumor cells that escape T cell attack. Memory NK cells are believed to function as potent effector cells in cancer immunotherapy. However, knowledge of their induction, identification, and potential in vivo is limited. Herein, we report on the induction and identification of memory-like NK cells via the action of a combination of a stimulator of interferon genes (STING) agonist loaded into lipid nanoparticles (STING-LNPs) and cytosine-phosphorothioate-guanine oligodeoxynucleotides (CpG-ODNs), and the potential of the inducted memory-like NK cells to prevent melanoma lung metastasis.

**Methods:**

The antitumor effects of either the STING-LNPs, CpG-ODNs, or the combination therapy were evaluated using a B16-F10 lung metastasis model. The effect of the combined treatment was evaluated by measuring cytokine production. The induction of memory-like NK cells was demonstrated via flow cytometry and confirmed through their preventative effect.

**Results:**

The combination of STING-LNPs and CpG-ODNs tended to enhance the production of interleukin 12 (IL-12) and IL-18, and exerted a therapeutic effect against B16-F10 lung metastasis. The combination therapy increased the population of CD11b^high^CD27^low^ NK cells. Although monotherapies failed to show preventative effects, the combination therapy induced a surprisingly strong preventative effect, which indicates that CD11b^high^CD27^low^ cells could be a phenotype of memory-like NK cells.

**Conclusion:**

As far as could be ascertained, this is the first report of the in vivo induction, identification, and confirmation of a phenotype of the memory-like NK cells through a prophylactic effect via the use of an immunotherapeutic drug. Our findings provide novel insights into the in vivo induction of CD11b^high^CD27^low^ memory-like NK cells thus paving the way for the development of efficient immunotherapies.

**Graphical Abstract:**

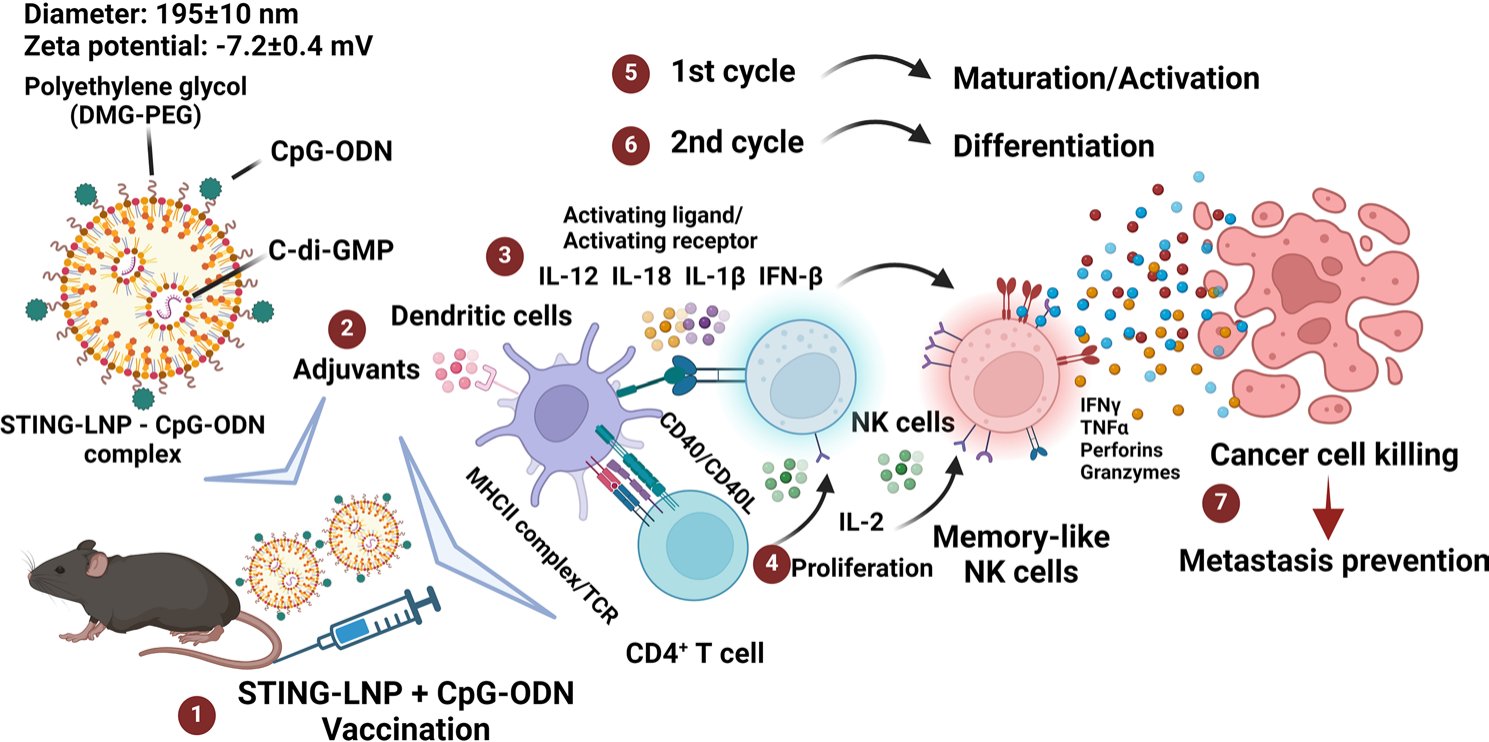

**Supplementary Information:**

The online version contains supplementary material available at 10.1186/s40164-024-00502-w.

## Background

Substantial efforts have recently been directed towards the advancement of tumor immune-based therapies that result in reducing the burden of tumors, extending patient survival, and inducing complete remission in some cases [1]. Most immunotherapies are T-cell based and are efficacious against many types of cancers [2–4]. However, because tumor cells can evade T-cell immune surveillance, this is considered a limitation to their effectiveness [5, 6]. Therefore, utilizing innate immune cells and empowering them to fight against cancer is critical, particularly in tumors that lack major histocompatibility complex class I (MHC-I) expression, which is considered to be the main cause of T-cell resistance [7, 8]. MHC-I expression is important for antigen presentation and, in turn, is indispensable for the effector function of T cells, but it is an inhibitory receptor for natural killer (NK) cells [9, 10]. Therefore, the loss of MHC-I expression in tumor cells is expected to allow NK cells to attack and kill the tumor cells.

Recent discoveries have led to a paradigm shift in the characterization of NK cells. Historically, NK cells were assumed to be short-lived cells associated with innate immune responses, whereas the existence of subsets of NK cells with memory functions has now been revealed. Although numerous issues remain to be addressed, NK cells with memory functions can be roughly divided into two types: memory NK cells and memory-like NK cells [11]. Memory NK cells found in viral infected models are specific to viral antigens [12]. Memory-like NK cells, however, are non-specific for certain antigens such as cytokine-induced memory-like (CIML) NK cells that have shown long-lasting effects even after the absence or discontinuation of a stimulus [13]. However, the potential of using CIML NK cells in cancer therapy in vivo is unclear, and available information concerning its phenotype remains limited.

Research over the past few decades has highlighted the great importance of innate immune memory in host defense as well as in the area of vaccinology [14, 15]. Memory-like NK cells were previously reported to be induced by Bacillus Calmette–Guérin (BCG). Accordingly, BCG is known to induce heterologous protection against various infections as well as many solid cancers [14, 16, 17]. In subsequent studies, researchers generated the memory-like features of natural killer cells through the use of a combination of cytokines that has resulted in enhanced functionality against many types of cancers [18, 19]. Many preclinical and very early stages of clinical trials have confirmed the safety and the promising functional activity of these CIML NK cells following their adoptive transfer [20–22]. Romee et al. reported that CIML NK cells could reduce the burden of tumors and enhance the survival of tumor-bearing mice [23]. In addition, complete remissions have been observed in patients with refractory acute myeloid leukemia following the infusion of CIML NK cells [20].

Researchers are currently attempting to identify the phenotype of these CIML NK cells. This memory phenotypical characteristic of NK cells differs depending on the nature of the induction and whether they are murine or human-derived [24]. Human CIML NK cells show an increase in CD94, NKG2D, NKG2A, NKp46, NKp44, and CD25 [20]. Murine CIML NK cells also show an increase in CD25 and CD11b [19]. In addition, adoptively transferred cytokine-preactivated murine NK cells display a mature phenotype of CD11b^high^CD27^low^, KLRG1^high^, and CD43^high^ with a potent effector function, but this phenotype has not yet been confirmed to be associated with memory responses [25].

For the effective induction of antitumor responses that are mediated by NK cells in vivo, we successfully developed a lipid nanoparticle (LNP) loaded with the stimulator of interferon genes (STING) agonist (STING-LNPs) [7, 8, 26–28]. The STING pathway is a cytosolic innate sensor for DNA and cyclic dinucleotides and is a potential candidate for use in cancer immunotherapy [7, 8]. STING-LNP treatment induced the production of systemic type I interferons (IFNs) leading to the activation of NK cells [8, 27, 29]. However, STING agonists do not efficiently induce the production of IL-12 and IL-18, which are indispensable for memory-like NK cell induction [30]. In contrast, CpG-ODN, an agonist of the toll-like receptor 9 (TLR9), is a potent adjuvant for inducing Th1 cytokine responses such as IL-12 and IL-18 [31]. Given the above information, we hypothesized that the use of a combination of STING-LNPs and CpG-ODNs might efficiently induce the production of CIML NK cells.

In the present study, we aimed to tackle the challenge of the in vivo induction of memory-like NK cells by utilizing the benefits of a proper delivery system. We investigated the potential of combining STING-LNPs with CpG-ODNs for the in vivo induction of the most mature and potent phenotype of NK cells, which we speculated would be CD11b^high^CD27^low^ NK cells. The combination therapy tended to enhance the production of IL-12 and IL-18 while significantly increasing the antitumor effect of the combination therapy against B16-F10 lung metastasis. Flow cytometry (FCM) analyses revealed that the combination therapy increased the most mature phenotype of NK cells, which were identified as CD11b^high^CD27^low^ NK cells. Two treatment cycles are essential for the induction of CD11b^high^CD27^low^ NK cells. Moreover, although monotherapies of either STING-LNPs or CpG-ODNs failed to show a preventative antitumor effect, the combination therapy induced a strong and long-term preventative antitumor effect because of the sustained presence of CD11b^high^CD27^low^ NK cells. As far as we could ascertain, this is the first report to confirm that the CD11b^high^CD27^low^ NK cells induced by the combination therapy of STING-LNPs and CpG-ODNs induces the phenotypical characteristics of CIML NK cells. These findings are the first to provide novel insights regarding the in vivo induction and the phenotypical characterization of CIML NK cells by using a combination of immunotherapeutic drugs that could not be achieved without the aid of a proper delivery system.

## Methods

### Reagents, cell lines, and mice

YSK12-C4, (6Z, 9Z, 28Z, 31Z)-19-(4-(dimethylamino) butyl) heptatriaconta-6,9,28,31-tetraen-19-ol, was synthesized as previously reported [32–36]. Cholesterol was obtained from Avanti Polar Lipids, Inc. (Alabaster, AL). The 1,2-dimirystoyl-sn-glycerol methoxyethyleneglycol 2000 ether (DMG-PEG_2000_) was purchased from the NOF Corporation (Tokyo, Japan). Cyclic di-GMP (c-di-GMP) was purchased from Cayman Chemical (Ann Arbor, MI). CpG-ODN (5’-ggGGTCAACGTTGAgggggg-3’, bases in lower letters are phosphonothioate) was synthesized by Hokkaido System Science (Sapporo, Japan). FITC anti-mouse CD3, PE anti-mouse NK1.1, PE/Cyanine7 anti-mouse KLRG1, APC anti-mouse CD27, Alexa700 anti-mouse CD11b, and APC/Cy7 anti-mouse CD43 antibodies and their isotype controls as well as the purified anti-mouse CD16/32 antibody and 7-AAD viability staining solution were purchased from Biolegend **(**San Diego, CA). A VersaComp Antibody capture bead kit was obtained from Beckman Coulter (Indianapolis, IN).

The B16-F10-luc2 cell line was purchased from Caliper Life Science (Hopkinton, MA). B16-F10-luc2 cells were cultured in RPMI-1640 (high glucose) containing 10% fetal bovine serum (FBS), 1 mM sodium pyruvate and 10 mM HEPES. C57BL/6 N female mice (6–7 weeks old) were purchased from Japan SLC Inc. (Shizuoka, Japan). All animal experiments were approved by the Ethics of Pharmaceutical Science Animal Care Committee of Hokkaido University (approval number: 20-01226).

### Preparation of the STING-LNPs

The STING-LNPs were prepared as previously reported [8]. In a typical preparation, 150 µL of 1 mM citrate buffer (pH 4.5) containing 500 nmol of c-di-GMP was added under vortexing to 300 µL of a lipid solution (YSK12-C4:cholesterol:DMG-PEG_2000_) = (85:15:1, mol ratio) in 90% t-butanol. The resultant mixture was added to 1.6 mL of 1 mM citrate buffer (pH 4.5) with vortexing followed by adding 4 mL PBS. This solution was filtered using an Amicon Ultra (MWCO 100,000) and was replaced with 4 mL of PBS. The particle size and zeta-potential of the resultant STING-LNPs was measured using a Zetasizer Nano ZS ZEN3600 (Malvern Instruments, UK). To assess the amount of c-di-GMP that was encapsulated, the STING-LNPs were lysed with 10% sodium deoxycholate, and the UV absorbance at 252 nm (ε = 24,700) was then measured via 264 UV/Vis spectroscopy. The required amounts of both the STING-LNPs and the CpG-ODNs were mixed immediately prior to injection. The resultant complex was characterized in a similar manner.

### Checking the cytokine levels in serum through ELISA

Mice were injected intravenously with either the STING-LNPs (4 µg), CpG-ODNs (150 µg), CpG-ODNs (250 µg), or the combination therapy (a mixture of STING-LNPs and CpG-ODNs). After 2 h, blood was collected, and the sera that was obtained was stored at −80°C. The concentrations of IFN-β, IL-12, IL-18, and IL-1β in the serum were quantified using an ELISA kit (R&D systems, USA) following the manufacturer’s instructions.

### Evaluating antitumor and prophylactic activity via luciferase assay

For the antitumor protocol, mice were challenged by B16-F10-Luc2 cells (2 × 10^5^) on day 0 to establish a pulmonary metastasis model. Following the intravenous administration of either PBS, STING-LNPs (4 µg), CpG-ODNs (250 µg), or combined therapies for two cycles on days 6 and 10, and the lungs were collected on day 14. In the case of the depletion experiment, either anti-NK1.1 or its isotype control clone C1.18.4 (200 µg) and either anti-CD8a or its isotype control clone LTF-2 (200 µg) was intraperitoneally injected on days 5 and 9 followed by the intravenous administration of either PBS or a combination of STING-LNPs and CpG-ODNs on days 6 and 10. On day 14, lungs were collected and used in luciferase activity measurements. For the prophylactic protocol, mice were intravenously administered PBS as a control and either the STING-LNPs (4 µg), CpG-ODNs (250 µg), or the combined therapies on days 0 and 4 for two cycles, and then the mice were inoculated with tumor cells either on days 5, 14, 24 or 90, after which lungs were collected on days 15, 28, 38, or 104, respectively. Tumor metastasis was assessed as previously reported [7, 8]. The lung samples were completely homogenized in lysis buffer, and luciferase activity was then quantified using a luminometer (Luminescencer-PSN, ATTO, Japan). In the case of the immune cell depletion experiment, the values of relative light units (RLU) per whole lung for the control group (PBS-treated mice) were set to 1.

### FCM analysis for evaluating NK cell markers in the spleen

Mice were challenged with B16-F10-Luc2 cells (2 × 10^5^) on day 0 to establish a pulmonary metastasis model. This was followed by intravenous administrations of either PBS, STING-LNPs (4 µg), CpG-ODNs (250 µg), or combined therapies on days 6 and 10 for two cycles. A single-cell suspension from the spleen was prepared for the FCM experiment on day 14. For the prophylactic protocol, mice were intravenously administered PBS as a control, and either the STING-LNPs (4 µg), CpG-ODNs (250 µg) or combined therapies on days 0 and 4 for two cycles, and tumor cells were inoculated either on days 5, 14, or 24 followed by spleen collection on days 15, 28, or 38, respectively. To track the induction and the functional lifetime of the memory-like NK cells, mice were intravenously administered PBS as a control or the combination of STING-LNPs (4 µg) and CpG-ODNs (250 µg) on days 0 and 4 for two cycles followed by spleen collection on days 7, 14, 21, and 28. To evaluate the effect of tumor cell inoculation on the induction level of memory-like NK cells, mice were intravenously administered either PBS as a control or the combination of STING-LNPs (4 µg) and CpG-ODNs (250 µg) on days 0 and 4 for two cycles, and tumor cells were inoculated on day 24 in one group of mice followed by spleen collection on day 28. Purified anti-mouse CD16/32 antibody was added to the splenocytes for blocking. The splenocytes were then stained with the FITC anti-mouse CD3 Antibody, the PE anti-mouse NK1.1 Antibody, the PE/Cyanine7 anti-mouse KLRG1 antibody, the APC anti-mouse CD27 antibody, the Alexa700 anti-mouse CD11b antibody, the APC/Cy7 anti-mouse CD43 antibody, and the isotype controls. The splenocytes were stained with 7-AAD and then analyzed using a CytoFlex (Beckman Coulter, Indianapolis, IN). Data were analyzed using FlowJo software.

### Statistical analysis

All statistical analyses were performed using GraphPad Prism software. Comparisons between the two treatments were performed using an unpaired t-test. Comparisons between multiple treatments were performed utilizing one-way analysis of variance (ANOVA) followed by a Tukey test. Statistical significance was designated as follows: ****p < 0.0001; ***p < 0.001; **p < 0.01; *p < 0.05.

## Results

### Combining the STING-LNPs with CpG-ODNs enhanced the production of cytokines, which was associated with the induction of CIML NK cells

The STING-LNPs were prepared using YSK12-C4 as the main component. YSK12-C4 is an ionizable lipid with a high affinity for immune cells and is well known for its high fusogenic activity that allows efficient cytosolic delivery [7, 8, 32, 33, 37]. The diameter, polydispersity index (PDI), and the zeta potential of the LNPs were 170 ± 15 nm, 0.048 ± 0.023, and 7.1 ± 0.4 mV, respectively (mean ± SD, *n* = 4). The required amounts of both STING-LNPs and CpG-ODNs were simply mixed and characterized before injecting the combination therapy. The diameter, PDI, and zeta potential of the complex was 195 ± 10 nm, 0.082 ± 0.021, and − 7.2 ± 0.4 mV, respectively (mean ± SD, *n* = 3). We screened two in vivo doses of CpG-ODNs for the induction of IFN-β, IL-12, IL-18, and IL-1β, essential cytokines for the activation of NK cells and their differentiation into CIML NK cells [19, 38]. Mice were intravenously administered either the STING-LNPs (4 µg), CpG-ODNs (150 or 250 µg), or the combination therapy. The monotherapy of the STING-LNPs drastically induced the induction of IFN-β. In contrast, the use of only CpG-ODNs induced only low levels. The use of a combination, however, induced much higher levels than that observed in the case of CpG-ODN monotherapy (Fig. S1A, B). The STING-LNP monotherapy failed to induce IL-12, and, in a similar manner, only a low level of IL-18 was observed. In addition, only low levels of IL-12 and IL-18 were induced by the administration of 150 µg of CpG-ODNs (Fig. S1B and C). However, the combination of the STING-LNPs and 250 µg of CpG-ODNs induced high levels of IL-12 and IL-18 (Fig. S1C and D). Furthermore, IL-1β was similarly induced by all groups (Fig. S1E). These results indicate the importance of combining CpG-ODNs with STING-LNPs for the induction of IL-12 and IL-18. Accordingly, 250 µg of CpG-ODNs combined with the STING-LNPs resulted in the highest level of induced IL-12, and appears to be a good candidate for the induction of CIML NK cells.

### The combination of STING-LNPs and CpG-ODNs stimulated the production of memory-like NK cells

**Fig. 1:**
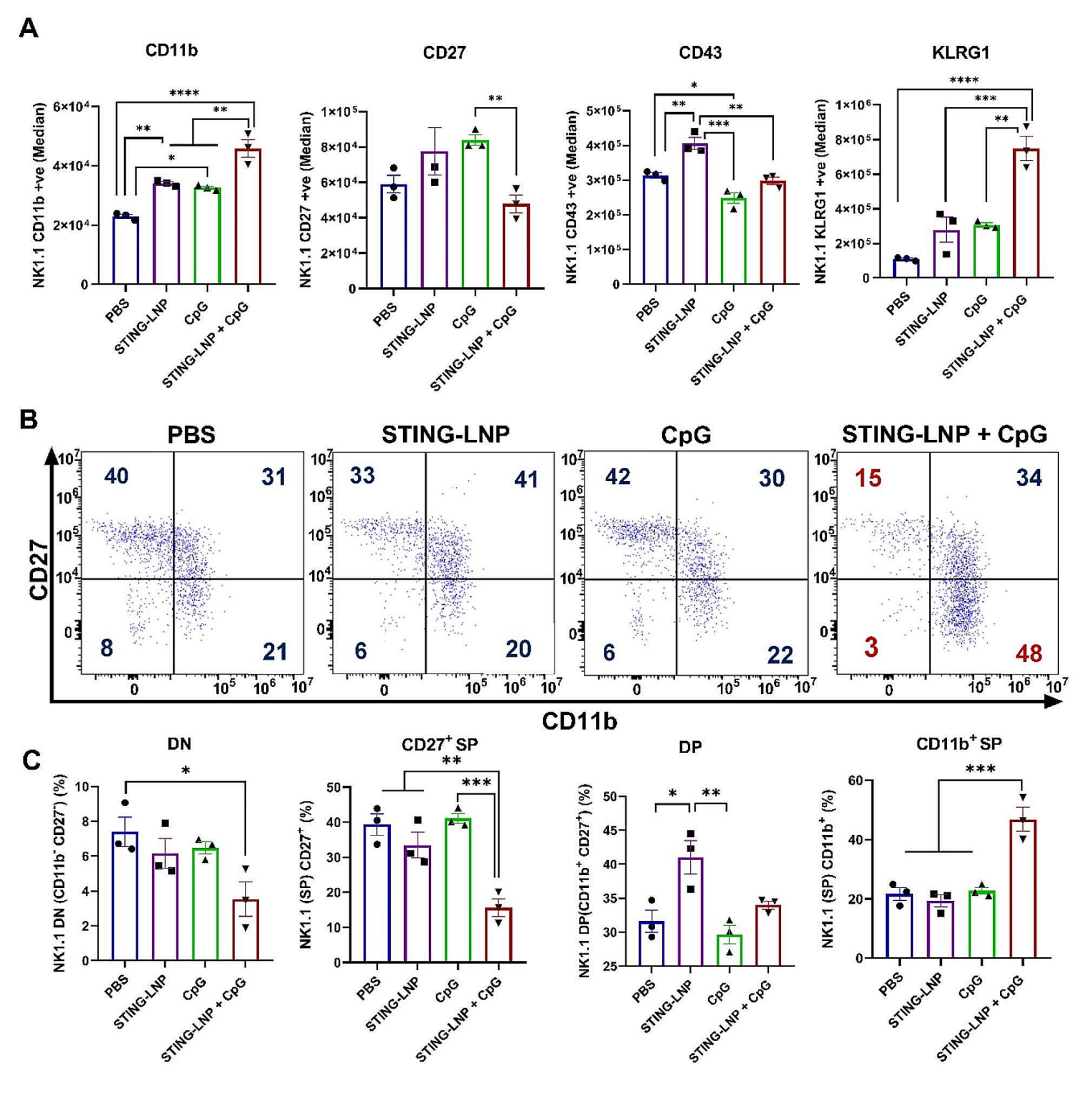
Combining STING-LNPs and CpG-ODNs can generate memory-like NK cells. Mice were intravenously injected with B16-F10-Luc2 cells (2 × 10^5^). PBS, STING-LNPs (4 µg/mouse of c-di-GMP), CpG-ODNs (250 µg), or the combination therapy was intravenously injected. (**A**) The FI (Median) of CD11b, CD27, CD43, and KLRG1 expression in NK cells. (**B**) Dot plots show the expressions of CD11b and CD27 on splenic NK cells (gated on CD3ε^−^NK1.1^+^) on day 14. Numbers indicate percentages. (**C**) The percentages of DN (CD11b^−^ CD27^−^), CD27^+^ SP (CD11b^−^ CD27^+^), DP (CD11b^+^ CD27^+^), and CD11b^+^ SP (CD11b^+^ CD27^−^) NK cells. The values represent the mean ± SEM (*n* = 3), ****p < 0.0001; ***p < 0.001; **p < 0.01; *p < 0.05). DN, double negative; SP, single positive; DP, double positive

Next, we confirmed our hypothesis that the combination therapy induces the activation of NK cells as well as promoting their differentiation into memory-like NK cells, which is the most mature phenotype of NK cells that are characterized as CD11b^high^ CD27^low^ NK cells. Mice with lung metastasis were intravenously administered either PBS, the STING-LNPs, CpG-ODNs, or the combination therapy for two cycles on days 6 and 10. The spleens were collected and checked for the activation and maturation of NK cells and to identify the presence of memory-like NK cells by FCM. After gating the NK cell population (NK1.1^+^CD3^−^), the expressions of CD11b, CD27, CD43, and KLRG1 were evaluated (Fig. S2). The combination of the STING-LNPs and CpG-ODNs resulted in a significant increase in the level of expression of CD11b as well as the percentage of CD11b^+^ NK cells by comparison with the other groups (Fig. 1A and S3). The level of CD27 expression and the percentage of CD27^+^ NK cells were significantly reduced following the combination therapy compared with that of the other groups (Fig. 1A and S3). In addition, the expression level of KLRG1 and the percentage of KLRG1^+^ NK cells were drastically increased after the combination therapy compared with that of the other groups (Fig. 1A and S3). These results indicate that the combination of the STING-LNPs and CpG-ODNs induces both the maturation and activation of NK cells. We also checked the induction of memory-like NK cells based on the simultaneous expressions of CD11b and CD27, because we speculated that producing NK cells with the most mature phenotype would lead to the generation of memory-like NK cells. The surface densities of CD27 and CD11b divide the murine NK cells into four subsets as follows: CD11b^−^ CD27^−^ cells (double negative: DN), CD11b^−^ CD27^+^ cells (single positive: CD27^+^ SP), CD11b^+^ CD27^+^ cells (double positive: DP), and CD11b^+^ CD27^−^ cells (CD11b^+^ SP). These four developmental stages from the most immature phenotype, have the ability to secrete cytokines and would have the best ability to secrete cytokines until the most mature and potent NK cells with the highest cytolytic function had developed [39]. The dot plots shown in Fig. 1B reveal that the NK cell population was changed drastically only after the combination therapy. The percentage of the DN population was comparable between all groups except for the combination group, which revealed a drastic decrease (Fig. 1C). In a similar manner, the percentage of the CD27^+^ SP population was comparable among all groups, but it was significantly reduced following the combination group (Fig. 1C). Moreover, the percentage of DP population was similar for all groups except for the STING-LNPs that showed a slight increase that was nonetheless comparable to that of the combination group (Fig. 1C). In addition, the percentage of the CD11b^+^ SP population was exclusively and drastically increased following the administration of the combination of the STING-LNPs and CpG-ODNs by comparison with that of all other groups (Fig. 1C). Though there was a drastic change in the subset of NK cells following the four treatment groups, there was no significant change in the percentage of NK cells (Fig. S4). These results confirm that the combination therapy causes an increase in the numbers of the most mature and functional stage of NK cells as CD11b^high^CD27^low^ with a high KLRG1 expression, which is related to the phenotype of the memory NK cells induced by a viral infection with KLRG1^high^ and CD27^low^ expression [12].

### NK cells play a significant role in the enhancement of the antitumor effect of the combination of STING-LNPs and CpG-ODNs

**Fig. 2:**
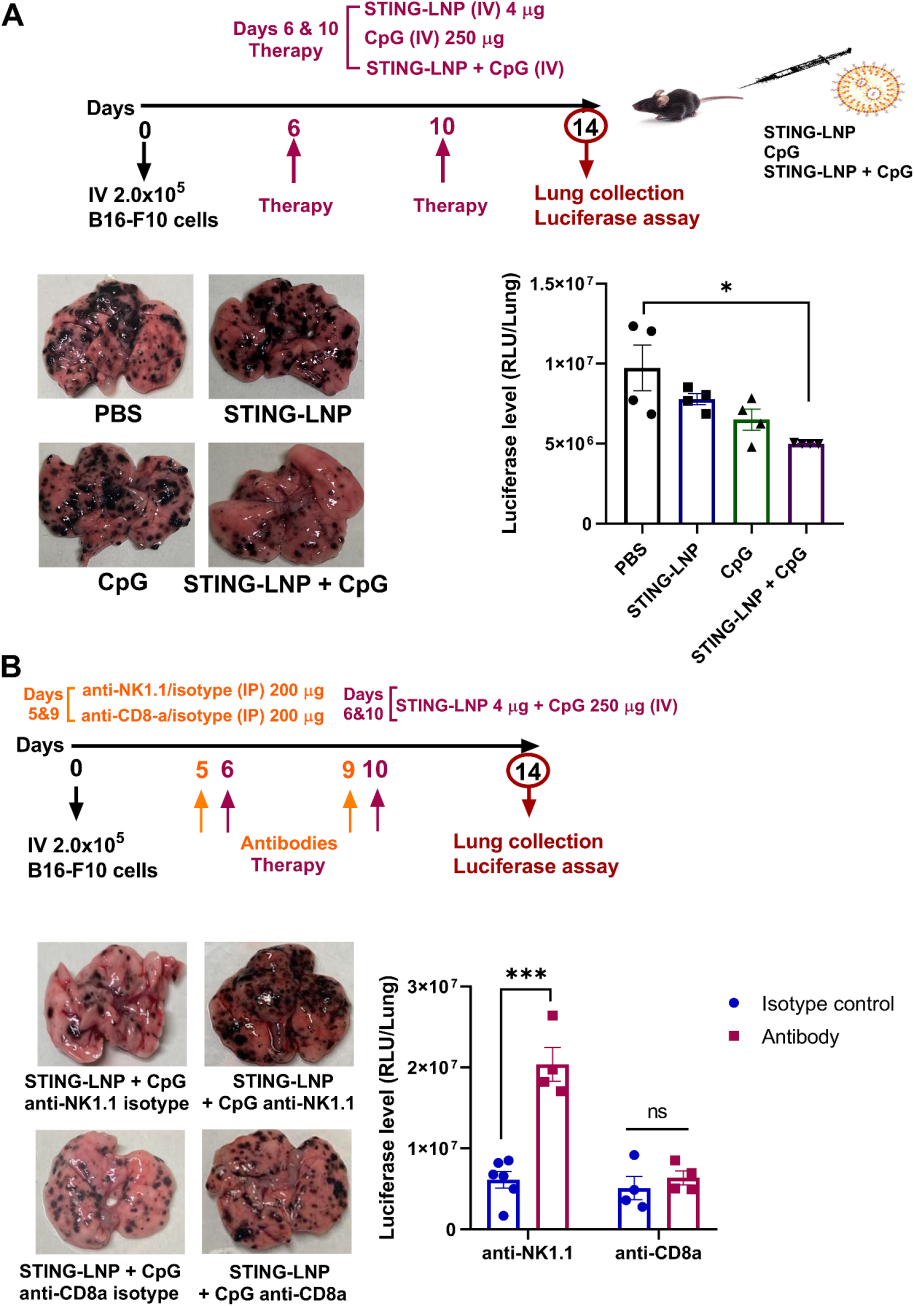
NK cells play a significant role in the enhancement of the antitumor effect of STING-LNPs and CpG-ODNs. (**A**) The antitumor effect of monotherapies and that of the combination therapy against B16-F10 lung metastasis. Mice were intravenously injected with B16-F10-Luc2 cells (2 × 10^5^). Either PBS, STING-LNPs (4 µg/mouse of c-di-GMP), CpG-ODNs (250 µg), or the combination therapy was intravenously injected for two cycles. The images represent the lungs of different treatment groups and the quantitative analysis of lung metastasis based on the values of luciferase activities. The values represent the mean ± SEM (*n* = 4, *P < 0.05). (**B**) The effect of the depletion of NK cells or cytotoxic T lymphocytes (CTL) on antitumor activity via the combination therapy. B16-F10 lung metastasis-bearing mice were intraperitoneally injected with either anti-NK1.1 (200 µg), anti-CD8a (200 µg), or their isotype control in similar doses. The images represent the lungs of different treatment groups and quantitative analysis of lung metastasis based on the values of luciferase activities. The RLU values per whole lung for the PBS-treated mice was set to 1. The values represent the mean ± SEM (*n* = 4–6, *P < 0.05). ns, non-significant

In the next step, we examined the ability of this combination therapy to induce therapeutic efficacy against a B16-F10 lung metastasis model. As shown in Fig. 2A, mice with lung metastasis were intravenously administered either PBS, the STING-LNPs, CpG-ODNs, or a combination of both for two cycles on days 6 and 10. Lungs were collected on day 14 to evaluate the antitumor effect. The combination therapy clearly caused a reduction in the number of tumor colonies in the lungs (Fig. 2A). In addition, the combination therapy resulted in a significant reduction in luciferase activity, indicating that the numbers of tumor cells had decreased (Fig. 2A). We subsequently examined the contribution of NK cells and CD8^+^ T cells on the antitumor effect via administration of the combination therapy. An anti-NK1.1 antibody treatment abrogated the antitumor effect via the combination therapy, whereas following the the anti-CD8a antibody treatment the antitumor effect was maintained (Fig. 2B). This indicates that the antitumor effect caused by the combination therapy is largely dependent on NK cells.

### Two treatment cycles are indispensable for achieving tumor reduction and CIML NK cell induction

**Fig. 3:**
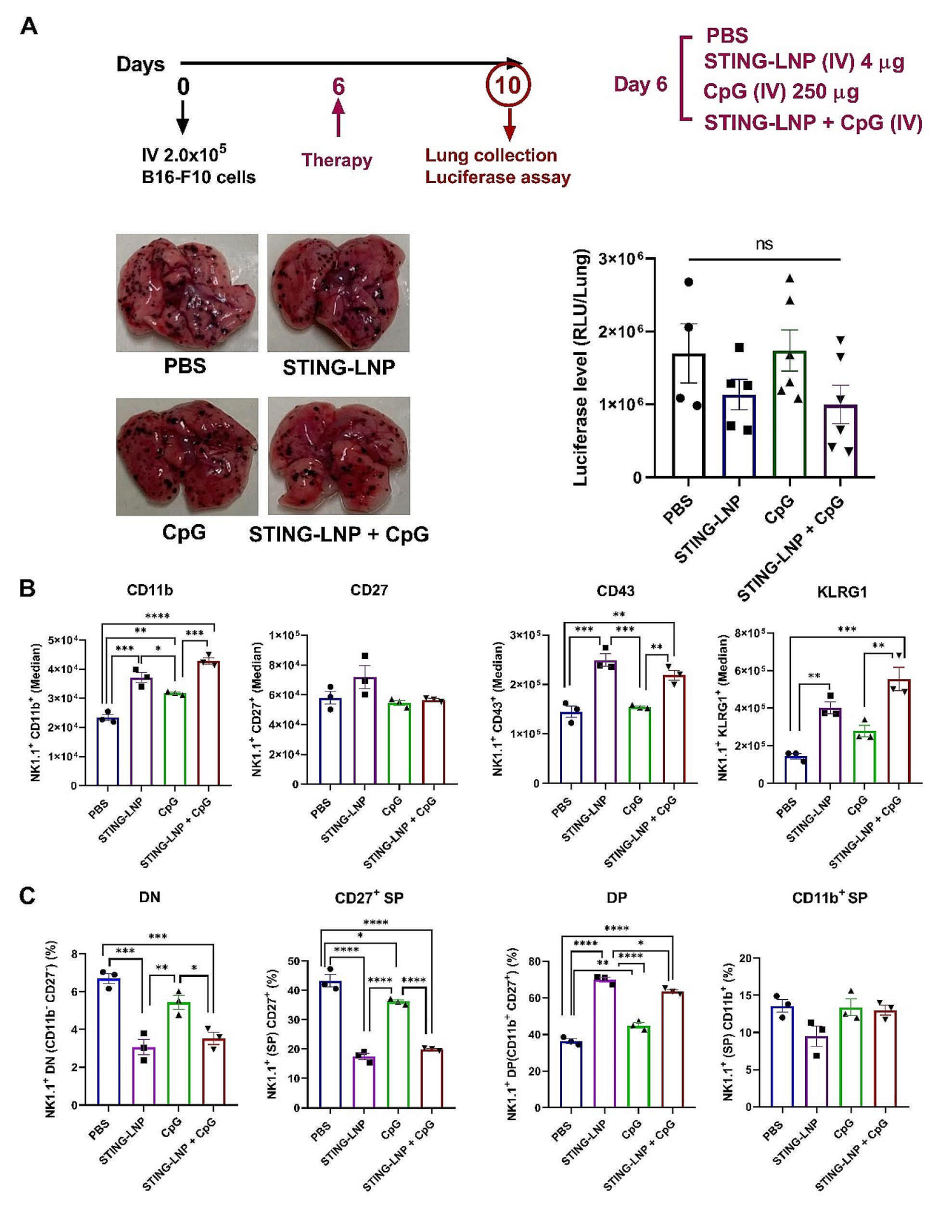
Two cycles of STING-LNPs and CpG-ODNs are indispensable for tumor reduction and memory-like NK cell induction. (**A**) Antitumor effects of monotherapies and combination therapy against B16-F10 lung metastasis. Mice were intravenously injected with B16-F10-Luc2 cells (2 × 10^5^). PBS, STING-LNPs (4 µg/mouse of c-di-GMP), CpG-ODNs (250 µg), or the combination therapy was intravenously injected for one cycle. Images show the lungs of different treatment groups, and the quantitative analysis of lung metastasis was based on the values of the luciferase activities. The values represent the mean ± SEM (*n* = 4–6). ns, non-significant. (**B**) The FI (Median) values for CD11b, CD27, CD43, and KLRG1 expression in NK cells. (**C**) The percentages of DN (CD11b^−^ CD27^−^), CD27^+^ SP (CD11b^−^ CD27^+^), DP (CD11b^+^ CD27^+^), and CD11b^+^ SP (CD11b^+^ CD27^−^) NK cells (gated on CD3ε^−^NK1.1^+^) on day 10. The values represent the mean ± SEM (*n* = 3, ****p < 0.0001; ***p < 0.001; **p < 0.01; *p < 0.05). DN, double negative; SP, single positive; DP, double positive

The issue arose as to whether two cycles of this combination is actually required to produce a significant antitumor effect and CIML NK cell induction, or whether one cycle would be sufficient. B16-F10 lung metastasis-bearing mice were intravenously administered either PBS, the STING-LNPs, CpG-ODNs, or the combination therapy for one cycle on day 6, and on day 10 the lungs were collected (Fig. 3A). Termination was performed on day 10, but not day 14, to fix the four-day interval between the treatment administration and termination as a two-cycle administration protocol. After the one-cycle administration, no significant reduction in tumor burden was observed, but the burden was reduced slightly following the administration of STING-LNPs and the combination therapy (Fig. 3A). We also examined the issue of whether or not one cycle of our combination therapy could activate NK cells and induce their differentiation to memory-like cells. Following a similar protocol, spleens were collected and the surface markers were checked via FCM. In the STING-LNPs and the combination therapy, the levels of expression of CD11b and KLRG1 and the percentages were drastically elevated and reached levels similar to that following two cycles of the combination therapy (Fig. 3B and S5). We then checked the simultaneous surface density of CD11b and CD27 on the NK cells. The percentages of DN and CD27^+^ SP populations were significantly reduced in the case of the STING-LNPs and the combination therapy (Fig. 3C). Additionally, the percentage of the DP population was drastically elevated following the administration of either STING-LNPs alone or the combination therapy (Fig. 3C). However, the percentage of the CD11b^+^ SP was comparable among all groups (Fig. 3C). Collectively, the percentages of DN and CD27^+^ SP populations were similar after one and two cycles of the combination therapy. Although DP was the highest population following one cycle, it failed to reach the population of CD11b^+^ SP that had been drastically increased following the two cycles (Fig. S6). These results indicate that one cycle of the combination therapy enhances the maturation and activation of NK cells, but show that it was insufficient for inducing the most potent phenotype of NK cells that express CD11b^high^CD27^low^, which we speculate are memory-like NK cells.

### Long-term vaccination effect through the sustained persistence of memory-like NK cells induced by the combination of STING-LNPs and CpG-ODNs

**Fig. 4:**
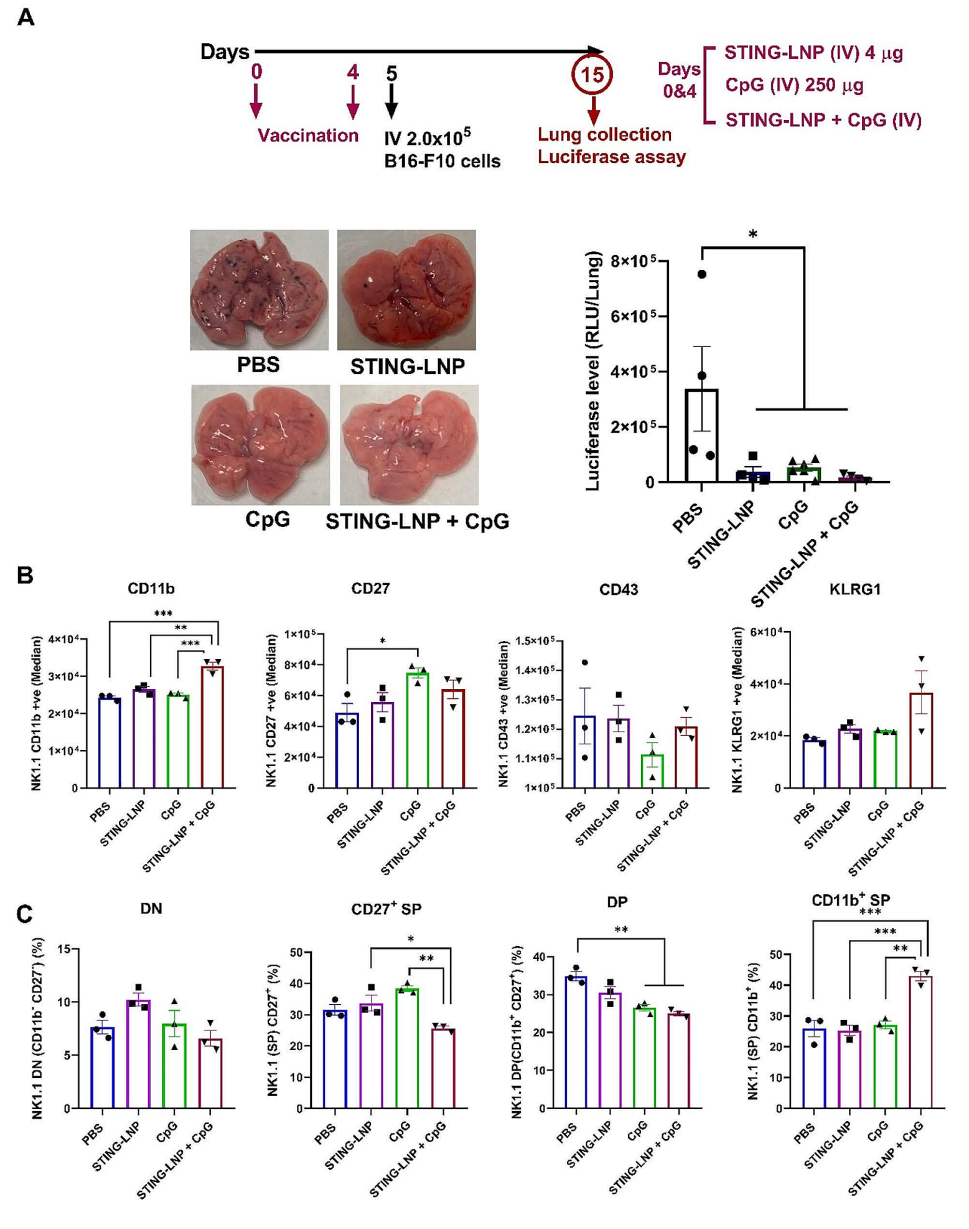
Antitumor effect based on the innate activation of NK cells. (**A**) Antitumor effect of monotherapies compared with that of the combination therapy against B16-F10 lung metastasis following a prophylactic protocol. Mice were intravenously injected with either PBS, STING-LNPs (4 µg/mouse of c-di-GMP), CpG-ODNs (250 µg), or the combination therapy for two cycles. After one day, the mice were intravenously injected with 2 × 10^5^ B16-F10-Luc2 cells. Representative photos show the lungs of the different treatment groups, and quantitative analysis of the lung metastasis was based on the values of luciferase activities. The values represent the mean ± SEM (*n* = 4–6, *P < 0.05). (**B**) The FI (Median) values for CD11b, CD27, CD43, and KLRG1 expression in NK cells. (**C**) The percentages of DN (CD11b^−^ CD27^−^), CD27^+^ SP (CD11b^−^ CD27^+^), DP (CD11b^+^ CD27^+^), and CD11b^+^ SP (CD11b^+^ CD27^−^) NK cells. The values represent the mean ± SEM (*n* = 3, ****p < 0.0001; ***p < 0.001; **p < 0.01; *p < 0.05). DN, double negative; SP, single positive; DP, double positive

Next, we examined the ability of combination therapy to induce a prophylactic effect against the B16-F10 lung metastasis model to confirm that CD11b^high^CD27^low^ NK cells are, in fact, memory-like NK cells. As shown in Fig. 4A, we employed a prophylactic protocol setting in which two cycles of either PBS, STING-LNPs, CpG-ODNs, or the combination therapy were intravenously administered on days 0 and 4, followed by tumor inoculation on day 5. All treatment groups resulted in a significant reduction in tumor burden (Fig. 4A). This finding suggests that the anti-tumor activity could be due to the instantaneous activation of an innate immune response and is independent of memory-like NK cell responses. Then, we checked the phenotype of the NK cells on day 15 following a similar protocol setting, but we used spleen samples instead. The level of expression of CD11b and the percentage of CD11b^+^ NK cells was significantly induced only by the combination therapy (Fig. 4B and Fig. S7). In addition, the percentage of CD27^+^ NK cells was significantly reduced only after administration of the combination therapy (Fig. S7). The expression and percentage of KLRG1 were elevated following administration of the combination group (Fig. 4B and S7). Then, we checked the simultaneous surface density of the CD11b and CD27 expression on the NK cells per group. The percentage of the DN population was similar among all groups except for a slight decrease for the combination group (Fig. 4C). The percentage of CD27^+^ SP was significantly reduced after administration of the combination therapy compared with that of each monotherapy (Fig. 4C). In addition, the percentage of the DP population was significantly decreased after the administrations of CpG-ODN and the combination therapy compared with that of the PBS group (Fig. 4C). Most importantly, the percentage of the CD11b^+^ SP population was drastically increased only for the group that received the combination therapy by comparison with that of all groups (Fig. 4C).

**Fig. 5:**
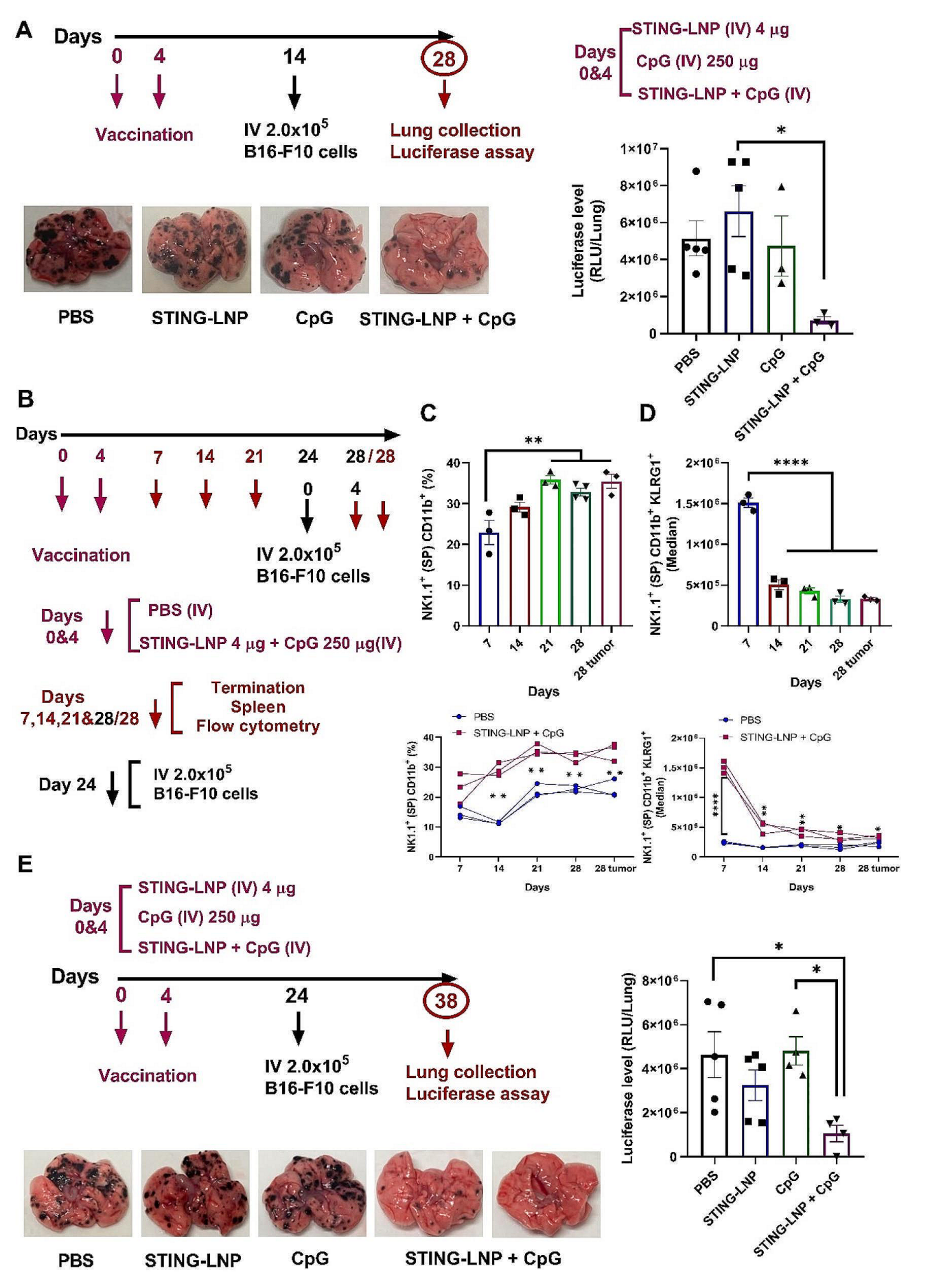
The sustained existence of the memory-like NK cells induced by the combination of STING-LNPs and CpG-ODNs resulted in prolonged protection against the development of melanoma lung metastasis. (**A**) The prophylactic effect of monotherapies compared with that of combination therapy against B16-F10 lung metastasis. Mice were intravenously injected with either PBS, the STING-LNPs (4 µg/mouse of c-di-GMP), CpG-ODNs (250 µg), or the combination therapy for two cycles. After 10 days, mice were intravenously injected with 2 × 10^5^ B16-F10-Luc2 cells. Representative photos show the lungs of the different treatment groups, and quantitative analysis of the lung metastasis was based on the values of luciferase activities. The values represent the mean ± SEM (*n* = 3–5, *P < 0.05). (**B**) The sustained persistence of the memory-like NK cells induced by STING-LNPs and CpG-ODNs vaccination. Mice were intravenously injected with either PBS or a combination of STING-LNPs (4 µg/mouse of c-di-GMP) and CpG-ODNs (250 µg) for two cycles. After 20 days one group of mice was intravenously injected with (2 × 10^5^) B16-F10-Luc2 cells. (**C**) The percentage of CD11b^+^ SP (CD11b^+^ CD27^−^) NK cells on the indicated days after vaccination. Upper graph: cell percentage among days for the SP CD11b^+^ NK vaccinated group. Bottom graph: comparison of the SP CD11b^+^ NK cell percentage of the vaccinated group with that of the PBS group on each termination day. The values represent the mean ± SEM (*n* = 3–4, **p < 0.01). (**D**) The FI (Median) of KLRG1 expression on CD11b^+^ SP (CD11b^+^ CD27^−^) NK cells on the indicated days after vaccination. Upper graph: comparison of the KLRG1 expression level of SP CD11b^+^ NK cells for the vaccinated group among days. Bottom graph: KLRG1 expression levels of SP CD11b^+^ NK cells comparing the PBS and vaccinated groups on each termination day. The values represent the mean ± SEM (*n* = 3, ****p < 0.0001; **p < 0.01; *p < 0.05). (**E**) Prolonged vaccination effect following two cycles of the STING-LNPs and CpG-ODNs. Mice were intravenously injected with either PBS, the STING-LNPs (4 µg/mouse of c-di-GMP), CpG-ODNs (250 µg), or the combination therapy for two cycles. After 20 days, mice were intravenously injected with B16-F10-Luc2 cells (2 × 10^5^). Representative photos show the lungs of different treatment groups, and quantitative analysis of the lung metastasis was based on the values of luciferase activities. The values represent the mean ± SEM (*n* = 4–5, *P < 0.05)

Accordingly, we changed the protocol setting to evaluate the memory function of the CD11b^high^CD27^low^ NK cells that were retained on day 15 after two cycles of vaccination. A prophylactic protocol was initiated, whereby two cycles of either PBS, the STING-LNPs, CpG-ODNs, or the combination therapy was administered intravenously on days 0 and 4 followed by tumor inoculation on day 14. The lungs were then collected on day 28, and luciferase activity among the groups was measured (Fig. 5A). Interestingly, only the combination therapy resulted in a dramatic preventative effect, while the other groups were comparable (Fig. 5A). This preventative effect confirmed that CD11b^high^CD27^low^ can be the phenotype of memory-like NK cells, and this was the first confirmation of the phenotype of the memory-like NK cells.

**Fig. 6:**
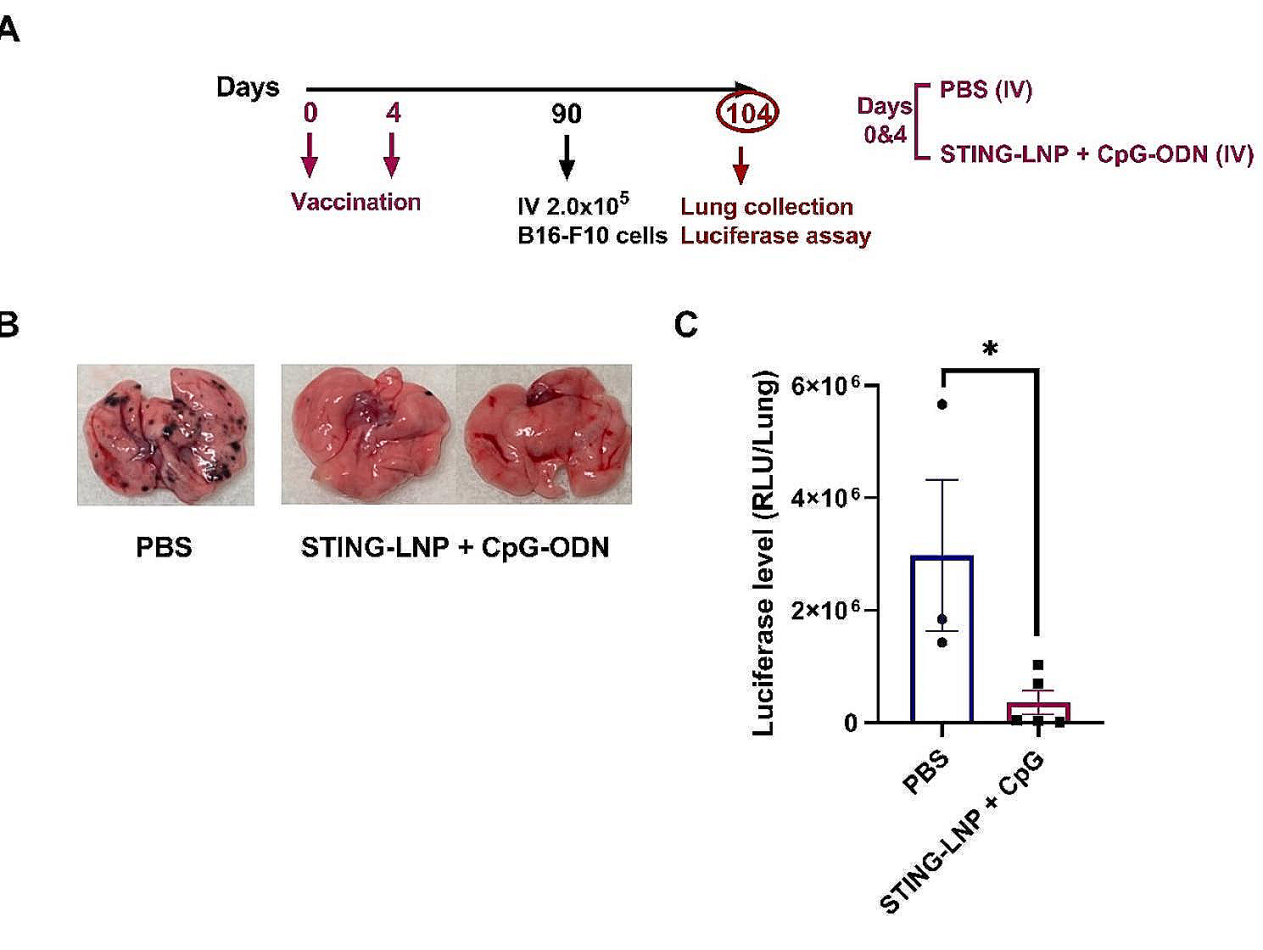
Effect of vaccination with the combination of STING-LNPs and CpG-ODNs against the development of melanoma lung metastasis after three months. (**A**) The prophylactic effect of the combination therapy against B16-F10 lung metastasis. Mice were intravenously injected with either PBS or the combination of STING-LNPs (4 µg/mouse of c-di-GMP) and CpG-ODNs (250 µg) for two cycles. After 90 days, mice were intravenously injected with 2 × 10^5^ B16-F10-Luc2 cells. (**B**) Representative photos show the lungs of different treatment groups, and (**C**) Quantitative analysis of the lung metastasis was based on the values of luciferase activities. The values represent the mean ± SEM (*n* = 3–5, *P < 0.05)

To track induction of the memory-like NK cells and their prolonged existence following the combination therapy with and without tumor inoculation, we employed a prophylactic protocol setting in which two cycles of either the PBS or the STING-LNPs and CpG-ODNs combination therapy was intravenously administered on days 0 and 4 (Fig. 5B). This was followed by checking the phenotype of the NK cells via FCM in the spleen on days 7, 14, 21, and 28. Other mice groups were challenged with B16-F10 cells on day 24 and the phenotype of the memory-like NK cells was checked at 4 days after inoculation. The population of SP CD11b^+^ NK cells was induced following the vaccination starting from day 7 and significantly in comparison to the PBS group on day 14. The SP CD11b^+^ NK cells reached a peak on day 21, and the level was maintained (Fig. 5C). Moreover, the expression of KLRG1, a differentiation marker, was tracked among the different days. The SP CD11b^+^ KLRG1^+^ expression level was drastically elevated at day 7 following the two-cycle vaccination when compared with all days of the PBS group (Fig. 5D). However (and unexpectedly), challenging mice with tumor cells did not affect the SP CD11b^+^ NK cells or the SP CD11b^+^ KLRG1^+^ expression levels (Fig. 5C and D). To examine the prolonged prophylactic effect of the extended lifetime of the memory-like NK cells induced by two cycles of the combination therapy, we employed a prophylactic protocol setting in which two cycles of either the PBS, the STING-LNPs, the CpG-ODNs, or the combination therapy was intravenously administered on days 0 and 4 followed by tumor inoculation on day 24. The lungs were then collected on day 38 and the luciferase activity among the groups was then measured (Fig. 5E). Only the combination of STING-LNPs and CpG-ODNs resulted in a significant preventative antitumor effect, and one sample even seemed to be completely protected against tumor induction (Fig. 5E). This prolonged prophylactic effect continued for 3 months following the vaccination (Fig. 6). These findings indicate that the sustained existence of CD11b^high^CD27^low^ memory-like NK cells induced by the administration of STING-LNPs and CpG-ODNs vaccination results in a prolonged prophylactic effect against lung metastasis.

## Discussion

The concept of inducing the production of memory-like NK cells for cancer immunotherapy has attracted the interest of a number of researchers. However, all the currently available research has involved either a pathogen-associated molecular pattern (PAMPS) or a combination of cytokines for the in vitro induction of the memory-like NK cells [16, 25]. Our findings are the first report concerning the in vivo induction and phenotypic characterization of memory-like NK cells showing a memory effect in cancer immunotherapy. The findings reported in the present study indicate that two cycles of the combination of STING-LNPs and CpG-ODNs are indispensable and tend to be sufficient for the release of cytokines that are required for the induction of memory-like NK cells that are, in turn, essential for the prevention of, and fight against, melanoma lung metastasis. Our findings have the potential for use in the development of immunotherapies that could harness the potential of NK cells for treating and preventing cancer metastasis.

For the development of efficient NK cell-based immunotherapy or vaccinations against cancers, an immune adjuvant that could induce the cytokines essential for memory-like NK cell induction is essential. STING activation results in the robust induction of type 1 IFNs but it also induces low levels of type-1 immune responses, in addition to its type 2 immune responses [8, 40]. On the other hand, CpG-ODNs are reported to induce high levels of type-1 immune responses, but IFNs are known to be only weakly induced [41, 42]. Therefore, to overcome this limitation, we combined the STING-LNPs and CpG-ODNs in order to induce type-1 immune responses, particularly those of IL-12, IL-18, and IFN-β. Concerning the mechanism responsible for how the STING-LNPs and CpG-ODNs induce the production of memory-like NK cells, trained immunity has been found to rely mainly on epigenetic and metabolic modifications. Epigenetic reprogramming includes DNA methylation, histone modification and chromatin opening for enhancing gene transcription and translation [43, 44]. These epigenetic changes are known to be inherited from parent to daughter cells [45]. Regarding the metabolic changes, the CIML NK cells tend to exhibit elevated expressions of nutrient transporters, and the metabolic profile is skewed towards glycolysis [44]. The glycolytic pathway is known to be essential for the IFN-γ and granzyme B production that achieves efficient levels of cytotoxicity [46]. In addition to blocking negative regulators such as killer immunoglobulin receptors (KIR) and to transforming growth factor-β (TGF-β) while enhancing the expression of CD25, IL-2 receptors are essential for the survival and proliferation of NK cells [47]. Accordingly, we expected that the first stimulus of this combination would activate myeloid cells to secrete cytokines that would then activate NK cells. Upon restimulation, the NK cells would acquire memory-like properties through epigenetic and metabolic changes that would result in a robust and quicker production of specific inflammatory cytokines and effector molecules thus harnessing the effect [44]. In addition to the previously mentioned mechanisms, we do believe that the delivery system utilized here plays a pivotal role in enhancing the induction of memory-like NK cells and effector molecules hence, the preventative effect. Thus, we plan to investigate the mechanism deeply via checking the genetic alteration in addition to the role of the delivery system.

CIML NK cells have shown enhanced antitumor responses particularly against melanoma cell lines [23]. The exact mechanism behind this is not yet completely understood. However, CIML NK cells were found to increase the production of IFN-γ, CD107a, and TNF [20, 23]. In addition, the upregulation of activating receptors such as NKG2D, NKP46, and DNAM-1 which are known to control NK cell cytotoxicity and cytokine production [20, 48]. The ligands of these activating receptors are reported to be present on the melanoma cell lines and allow for cytokine induction and degranulation in response to melanoma targets [49]. Moreover, memory-like NK cells can block inhibitory regulatory receptors and can even negate their signals [20, 47]. We therefore conclude that once the resting and self-renewing memory-like NK cells are sensitized by tumor cells, they will have the capacity to kill them, and, hence, prevent their metastasis and induction.

Herein we provide evidence that two cycles rather than one cycle of STING-LNPs and CpG-ODNs is required to induce the production of memory-like NK cells that are able to produce a significant antitumor effect. Our findings are consistent with previous reports showing that the first stimulus results in the activation of innate immune cells followed by a resting state until the second stimulus occurs, which is essential for the induction of trained immune cells [50]. In addition, Romee et al. reported that the functional activity of in vitro IL-12 + IL-18-preactivated NK cells was significantly increased compared with control NK cells only after restimulation [22]. Our previous report also showed that two cycles of STING-LNPs and anti-Programmed cell death-1 (PD-1) antibody were essential and sufficient for inducing an antitumor effect [7]. Our findings show that two cycles, rather than one cycle, of the combination therapy could result in significant tumor control following the induction of memory-like NK cells. One cycle of the STING-LNPs and that of the combination therapy induced only slight tumor control (Fig. 3A). Mechanistic findings have shown high expression levels of CD11b, CD43, and KLRG1 indicating that a higher maturation and activation of the NK cells occurred following one stimulus with the STING-LNPs and that of the combination group (Fig. 3B). Although one cycle of either the STING-LNPs or the combination therapy allowed NK cells to differentiate and reach the DP population, a CD11b^+^ SP population (memory-like NK cells) could not be achieved (Fig. 3C). This confirms the importance of the administration of two cycles of the STING-LNPs and CpG-ODNs for the induction of CD11b^high^CD27^low^ memory-like NK cells and hence, good tumor control. If the restimulation of innate immune cells could provide enhanced responses compared with the initial stimulus, reports have shown that the cells are considered to have acquired trained immunity [51].

Long-lived memory or memory-like NK cells have been reported, but the prolonged effect of the persistent memory-like NK cells has not yet been confirmed. Previously, because of murine cytomegalovirus (MCMV), memory NK cells were proven to have long lifetimes of around 90 days [12]. In addition, the in vitro preactivated CIML NK cells could still be detected after 3 weeks following its adoptive transfer [52]. Even though their lifetimes had been prolonged, these memory or memory-like NK cells were detected in very low percentages compared with their initial induction [12, 18]. However, we report herein that memory-like NK cells, induced in vivo through the combination of immunotherapeutic drugs, persist for at least one month at their peak percentage, with no reduction, which results in a prolonged prophylactic effect (Fig. 5C). The long-term persistence of the memory-like NK cells is controlled mainly by metabolic and epigenetic changes that are induced by the cytokines in CIML NK cells. IL-12, IL-18, and IL-15 are known to cause CIML NK cells to undergo a metabolic shift toward glycolysis that persists even after cytokine removal. This results in an enhancement of the proliferation, long-term persistence, and recall function of NK cells [53]. In addition, IL-15 and IL-2 play an important role in the proliferation, homeostasis, survival, expansion, and persistence of NK cells [54]. Terren et al. reported that the effector function of CIML NK cells, IFN-γ production, was greatly impaired following the inhibition of glycolysis [54]. In a similar manner, effects such as the proliferation and cytotoxicity of NK cells were impaired in vitro when glucose metabolism was inhibited, which resulted in susceptibility to MCMV infection in vivo [46]. Furthermore, previous studies have shown that memory-like NK cells have stability and heritable properties [18, 45, 55]. In other words, differentiation to memory-like NK cells through permanent or heritable changes at the epigenetic or metabolic level could result in a self-renewing population of cells regardless of a continuous stimulus [18]. Therefore, our findings concerning the prolonged persistence of the memory-like NK cells and the long-term vaccination effect that maintains their enhanced functionality could be related to their heritable properties.

Vaccines have historically shown great efficacy in preventing infections and/or decreasing their onset. Many vaccinations are available for the prevention of a variety of viral and bacterial infections, but researchers have struggled to develop cancer vaccines. The concept of trained immunity encouraged us to undertake this research and it promises to pave the way for the further development of prophylactic cancer vaccines. Vaccination with BCG has been reported to decrease the susceptibility towards many infections through the long-term boosting of the formation of innate immune cells. BCG was recently reported to protect against many cancers as well as COVID-19 through the induction of memory-like NK cells [56, 57]. Moreover, recent studies have shown that re-engineering the BCG with a STING-agonist-augmented trained immunity and enhanced the antitumor effect [58, 59]. Despite all these positive results regarding controlling the tumor burden through trained immunity, little effort has been directed towards developing a prophylactic tumor vaccine that could protect against cancers. We demonstrate herein that the combination of STING-LNPs and CpG-ODNs has offered protection against a melanoma lung metastasis model through inducing the formation of memory-like NK cells and extending their existence (Figs. 5 and 6). This drastic control of tumor metastasis and its prevention confirms the induction and the phenotypical characterization of memory-like NK cells as CD11b^high^CD27^low^ and KLRG1^high^ NK cells.

These findings promise to pave the way for the development of many efficient cancer immunotherapies by harnessing the potential of producing memory-like NK cells in vivo instead of via in vitro pre-activation and adoptive transfer. The phenotypical characterization of the memory-like NK cells performed and confirmed here will allow for easier identification later. In addition, the in vivo induction of memory-like NK cells will help in avoiding the consequences that sometimes occur during and after the infusion of memory NK cells to patients in clinical trials. Out of 13 patients in a clinical trial, 3 deaths were reported after infusion because of bacteremia with septic shock [20]. In addition, it may also protect against the inflammation that can occur towards the presence of foreign cells. Collectively, the in vivo induction of memory-like NK cells promises to be much safer than adoptive transfer as well as saving time and lowering costs.

## Conclusions

The findings of the present study provide novel insights regarding the in vivo induction and the phenotypic characterization of memory-like NK cells as CD11b^high^CD27^low^ and KLRG1^high^ NK cells through the use of a combination of immune adjuvants. These results highlight the importance of such cells in protecting against lung metastasis. In this study, two cycles of a combination of STING-LNPs and CpG-ODNs induced sufficient levels of cytokines that are essential for the activation and differentiation of NK cells to memory-like CD11b^+^ SP NK cells, which could have extended lifetimes. This resulted in an enhanced antitumor effect and protection against the development of a lung metastasis model.

### Electronic supplementary material

Below is the link to the electronic supplementary material.

Supplementary Material 1

## Data Availability

The datasets used and/or analyzed during the current study are available from the corresponding author on reasonable request.

## Abbreviations

STING: Stimulator of interferon genes
LNP: Lipid nano particle
IL: interleukin
MHC-I: Major histocompatibility complex-I
CIML: Cytokine induced memory-like
BCG: Bacillus Calmette Guerin
PD-1: Programmed cell death-1
FCM: Flow cytometry
DMG-PEG_2000_: 1,2-dimirystoyl-sn-glycerol methoxyethyleneglycol
C-di-GMP: Cyclic di-GMP
PBS: Phosphate Buffered Saline
CTL: Cytotoxic T lymphocyte
RLU: Relative light units
DN: Double negative
SP: Single positive
DP: Double positive
KIR: Killer immunoglobulin receptor
TGF-β: Transforming growth factor-β
MCMV: Murine cytomegalovirus
