# Supplementary material for: Vaccination with a combination of STING agonist-loaded lipid nanoparticles and CpG-ODNs protects against lung metastasis via the induction of CD11bhighCD27low memory-like NK cells
Source: Exp Hematol Oncol. 2024 Mar 29;13:36. doi: 10.1186/s40164-024-00502-w (PMC10981311; doi:10.1186/s40164-024-00502-w)
Supplement: Supplementary file 1 — Supplementary Material 1 [file 40164_2024_502_MOESM1_ESM.docx]

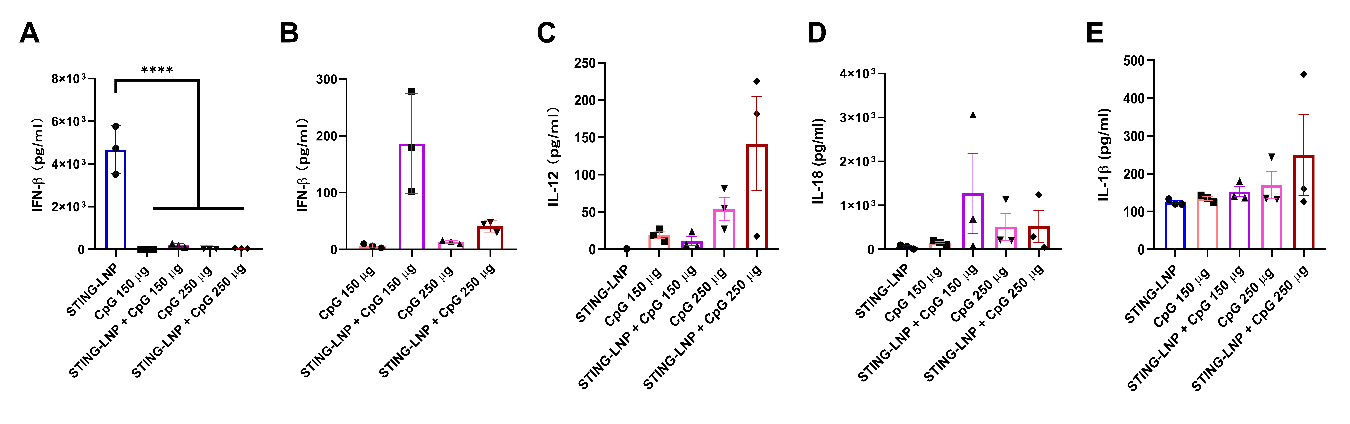


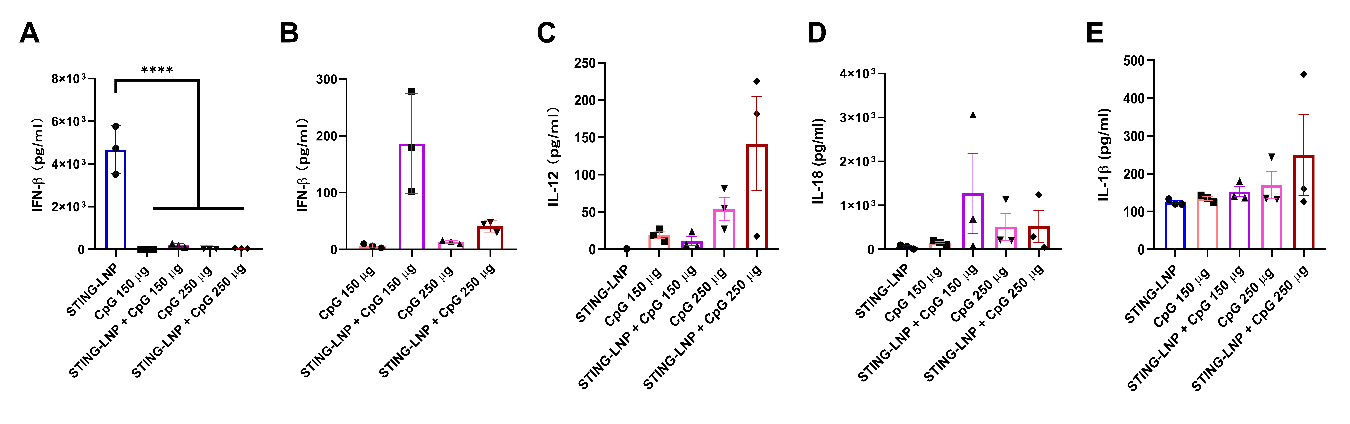


**Fig. S1. Combining STING-LNPs with CpG-ODNs enhances the production of cytokines**

**(A)** IFN-β concentration in serum (all groups). **(B)** IFN-β concentration in serum (without STING-LNPs). **(C)** IL-12 concentration in the serum. **(D)** IL-18 concentration in the serum. **(E)** IL-1β concentration in the serum. Mice were intravenously injected with the STING-LNPs (4 μg/mouse of c-di-GMP), CpG-ODNs (250 µg) or a combination of both. Blood samples were collected after 2 hours. Each cytokine concentration in the sera was measured via ELISA. The values represent the mean ± SEM (n = 3, ****p<0.0001).


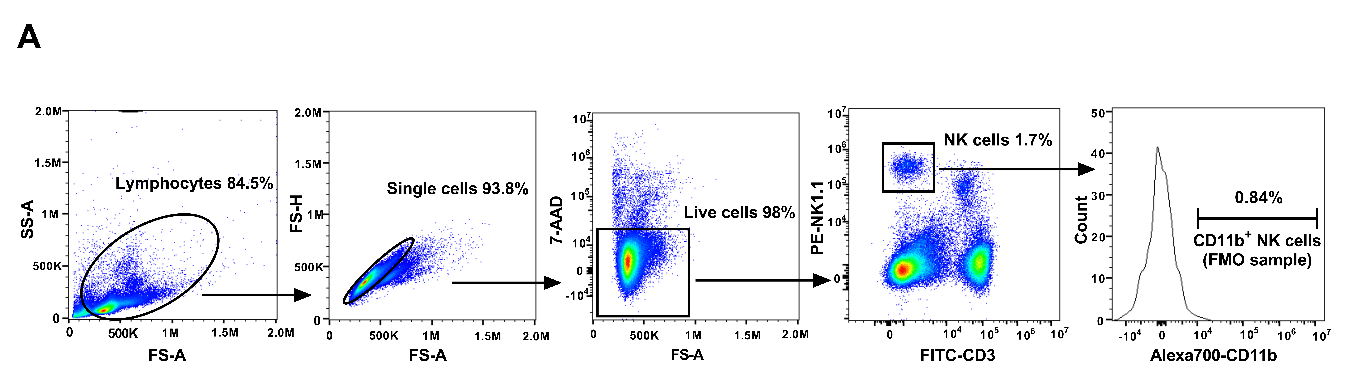


**Fig. S2. Gating strategy for FCM analysis in the spleen**

After gating live cells and lymphocytes, NK cells were identified as CD3^-^NK1.1^+^ cells. The CD11b^+^ gate, the CD27^+^ gate, the CD43^+^ gate, and the KLRG1^+^ gate were set via isotype control.

**
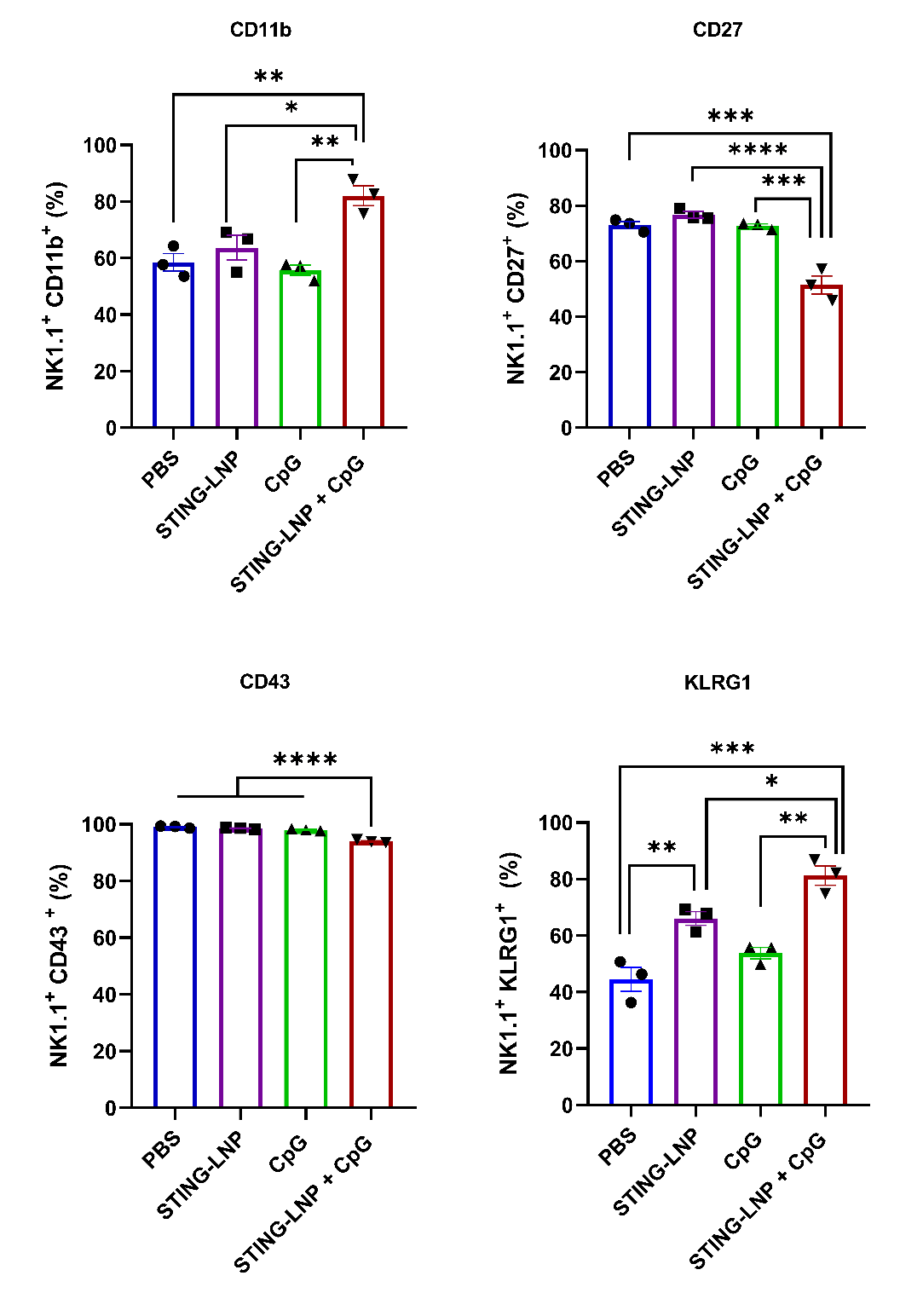
**

**Fig. S3.** **Combining STING-LNPs and CpG-ODNs induces the maturation and activation of NK cells**

Mice were intravenously injected with 2 × 10^5^ of B16-F10-Luc2 cells. PBS, STING-LNPs (4 μg/mouse of c-di-GMP), CpG-ODNs (250 µg), or a combination of both were intravenously injected. The percentages of CD11b^+^ NK cells, CD27^+^ NK cells, CD43^+^ NK cells, and KLRG1^+^ NK cells in the spleen. The values represent the mean ± SEM (n = 3, ****p<0.0001; ***p<0.001; **p<0.01; *p<0.05).


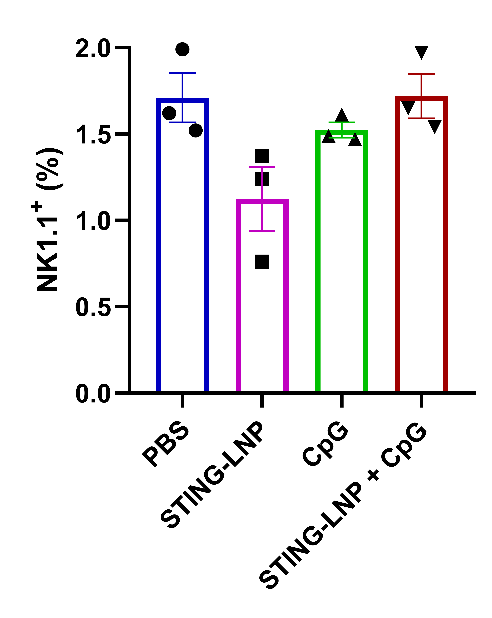


**Fig. S4. NK cell percentage among the four treatment groups**

Mice were intravenously injected with 2 × 10^5^ of B16-F10-Luc2 cells. PBS, STING-LNPs (4 μg/mouse of c-di-GMP), CpG-ODNs (250 µg), or a combination of both were intravenously injected. The percentages of NK1.1 cells in the spleen. The values represent the mean ± SEM (n = 3).


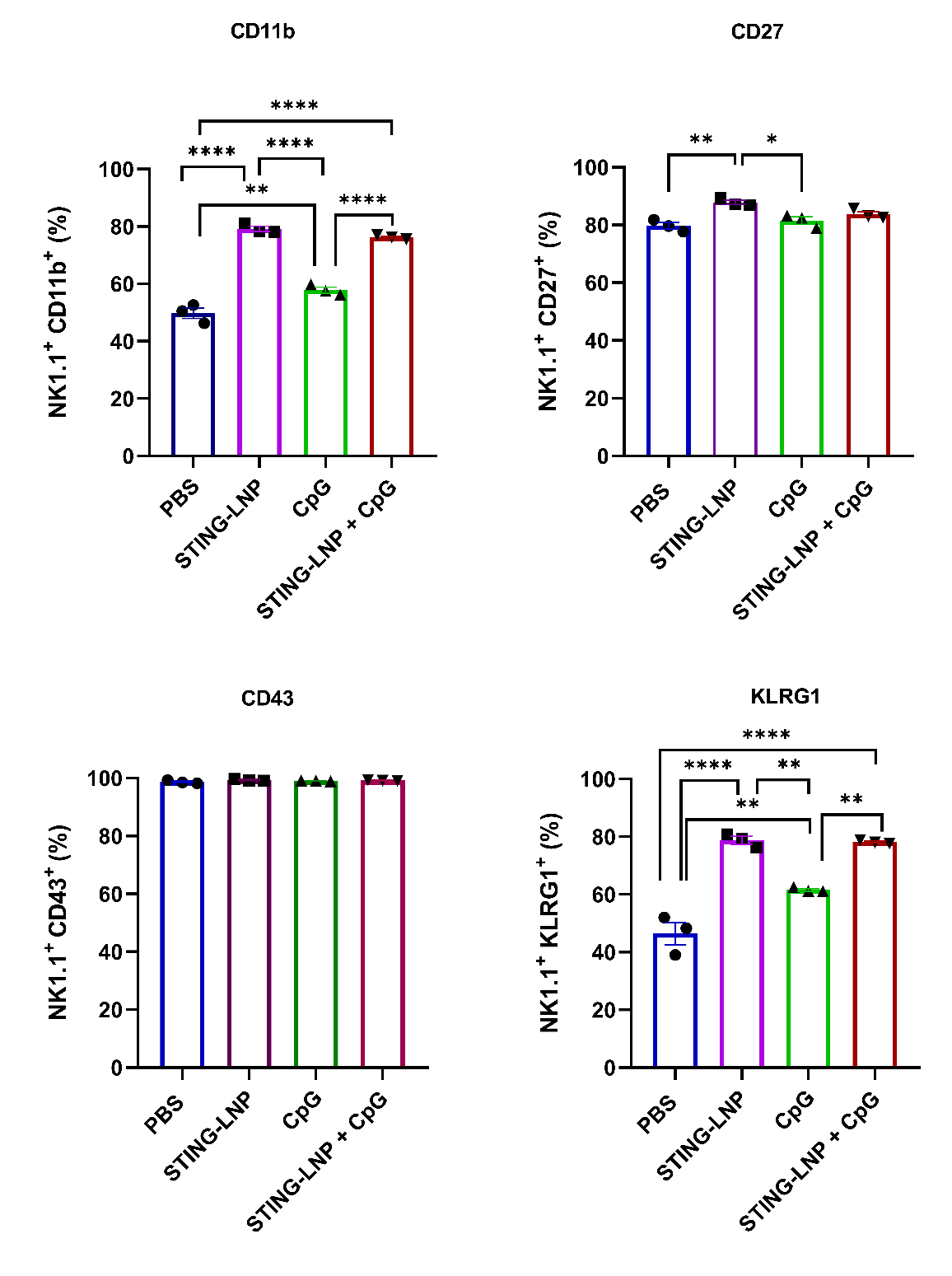


**Fig. S5. One cycle of combination therapy induces the maturation and activation of NK cells**

Mice were intravenously injected with 2 × 10^5^ of B16-F10-Luc2 cells. PBS, STING-LNPs (4 μg/mouse of c-di-GMP), CpG-ODNs (250 µg), or a combination of both were intravenously injected. The percentages of CD11b^+^ NK cells, CD27^+^ NK cells, CD43^+^ NK cells, and KLRG1^+^ NK cells in the spleen. The values represent the mean ± SEM (n = 3, ****p<0.0001; **p<0.01; *p<0.05).


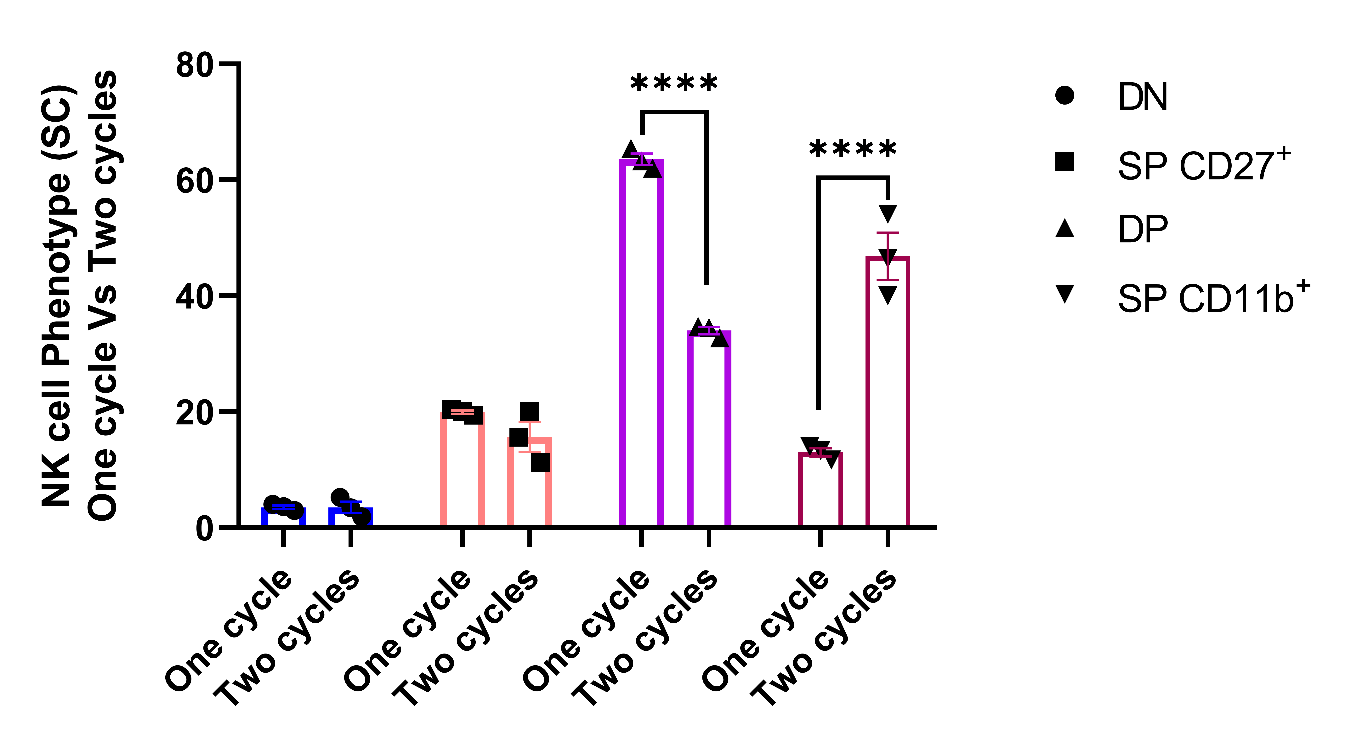


**Fig. S6. Two cycles are indispensable for the induction of memory-like NK cells**

A collective chart illustrating the differences between the four developmental stages of NK cells following one and/or two cycles of STING-LNP and CpG-ODN administration. Mice were intravenously injected with 2 × 10^5^ B16-F10-Luc2 cells. PBS, STING-LNPs (4 μg/mouse of c-di-GMP), CpG-ODNs (250 µg), or a combination of both were intravenously injected. The percentages of DN (CD11b^-^ CD27^-^), CD27^+^ SP (CD11b^-^ CD27^+^), DP (CD11b^+^ CD27^+^), and CD11b^+^ SP (CD11b^+^ CD27^-^) NK cells (gated on CD3ε^−^NK1.1^+^). The values represent the mean ± SEM (n = 3, ****p<0.0001). DN, double negative; SP, single positive; DP, double positive; SC, STING-LNP + CpG-ODN; Vs, versus.

**
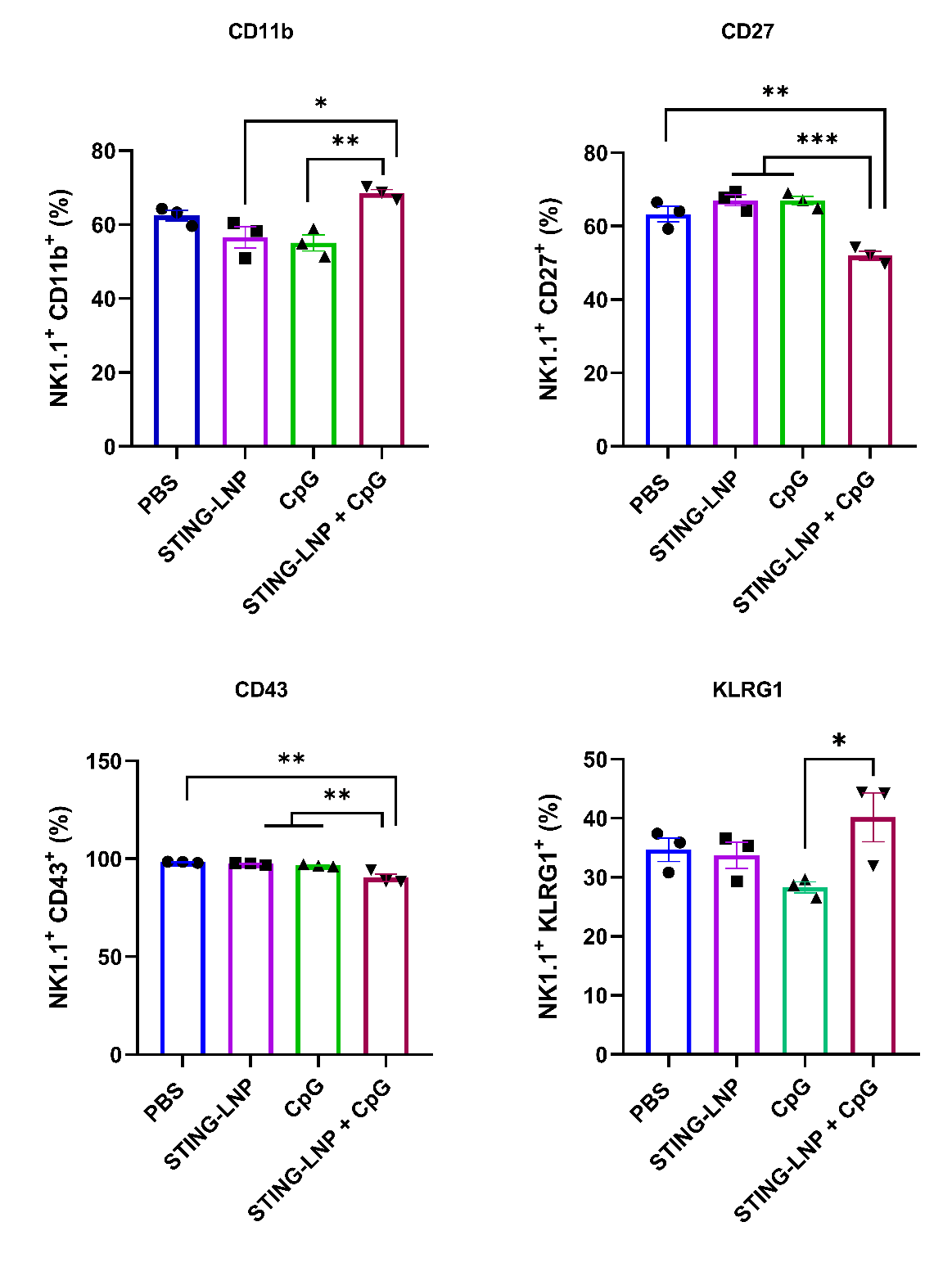
**

**Fig. S7. Prophylactic administration of STING-LNPs and CpG-ODNs induce the activation and maturation of NK cells**

PBS, STING-LNPs (4 μg/mouse of c-di-GMP), CpG-ODNs (250 µg), or a combination of both were intravenously injected followed by intravenous injection of the vaccinated mice with 2 × 10^5^ of B16-F10-Luc2 cells. The percentages of CD11b^+^ NK cells, CD27^+^ NK cells, CD43^+^ NK cells, and KLRG1^+^ NK cells in the spleen. The values represent the mean ± SEM (n = 3, ***p<0.001; **p<0.01; *p<0.05).
